# EVs-miRNA: The New Molecular Markers for Chronic Respiratory Diseases

**DOI:** 10.3390/life12101544

**Published:** 2022-10-05

**Authors:** Piera Soccio, Giorgia Moriondo, Donato Lacedonia, Pasquale Tondo, Carla Maria Irene Quarato, Maria Pia Foschino Barbaro, Giulia Scioscia

**Affiliations:** 1 Department of Medical and Surgical Sciences, University of Foggia, 71122 Foggia, Italy; 2 Institute of Respiratory Diseases, Policlinico Riuniti of Foggia, 71122 Foggia, Italy

**Keywords:** extracellular vesicles, miRNA, chronic respiratory diseases, COPD, asthma, sleep disorders, IPF

## Abstract

Idiopathic pulmonary fibrosis (IPF), chronic obstructive pulmonary disease (COPD), asthma and sleep disorders are chronic respiratory diseases that affect the airways, compromising lung function over time. These diseases affect hundreds of millions of people around the world and their frequency seems to be increasing every year. Extracellular vesicles (EVs) are small-sized vesicles released by every cell in the body. They are present in most body fluids and contain various biomolecules including proteins, lipids, mRNA and non-coding RNA (micro-RNA). The EVs can release their cargo, specifically micro-RNAs (miRNAs), to both neighboring and/or distal cells, playing a fundamental role in cell–cell communication. Recent studies have shown their possible role in the pathogenesis of various chronic respiratory diseases. The expression of miRNAs and, in particular, of miRNAs contained within the extracellular vesicles seems to be a good starting point in order to identify new potential biomarkers of disease, allowing a non-invasive clinical diagnosis. In this review we summarize some studies, present in the literature, about the functions of extracellular vesicles and miRNAs contained in extracellular vesicles in chronic respiratory diseases and we discuss the potential clinical applications of EVs and EVs-miRNAs for their possible use such as future biomarkers.

## 1. Introduction

Chronic respiratory diseases represent an important health problem both from an epidemiological point of view (high mortality and morbidity) and for the disabling consequences and high health costs [1,2].

The World Health Organization (WHO) estimates that 4.6 million people die prematurely each year from chronic respiratory diseases, thus accounting for 5% of global deaths [1].

Chronic respiratory diseases arise due to the action of a set of risk factors: in most cases the main cause is the genetic predisposition linked to familial ties. However, other risk factors can be environmental: allergens, pollen and mites, but also tobacco smoke, work activities, occupational exposure, air pollution, social conditions and obesity turn out to be aggravating circumstances [3].

The main chronic respiratory diseases are asthma, chronic obstructive pulmonary disease (COPD), sleep disorders and idiopathic pulmonary fibrosis (IPF) [1,3].

Although effective preventive measures exist, chronic respiratory diseases are often underestimated, underdiagnosed, undertreated and insufficiently prevented. To date, in fact, the management of these diseases represents a significant health challenge that requires a diagnosis as early as possible, with standardized tools which must be followed by timely and appropriate therapies, capable of preventing or delaying disability and treating the chronically ill. Extracellular vesicles (EVs) are small vesicles coated with a phospholipid bilayer produced by many cell types capable of delivering their cargo contents to target cells. They have dimensions that can vary in the order of micro/nano and can be found in most biological fluids. To date, we know that EVs represent important mediators of intercellular communication, both in prokaryotes and eukaryotes, and that they are released by cells into the extracellular environment. These vesicles are capable of transferring biological signals between cells, influencing the physiological and pathological functions of the recipient cells [4,5].

EVs can transfer proteins, peptides, lipids and nucleic acids to target cells. In particular, the presence of small non-coding RNA molecules, microRNAs, which play a significant role in the regulation of gene expression, in cell–cell communication and in the modulation of protein synthesis, has been demonstrated, influencing the various cellular functions [6].

The extracellular vesicles offer numerous advantages in the delivery of microRNAs (miRNAs); in fact, they allow their long-distance transfer, and protect them from the degradation enzymes present at extracellular level [7].

The EVs, therefore, represent real vehicles able to package a message, the microRNAs, and to transmit it from a donor cell to a recipient cell. The EVs and microRNAs contained within these vesicles are currently the focus of multiple scientific studies aimed at understanding the dynamics of cell–cell communication. In particular, they have generated considerable interest in clinical applications, as diagnostic biomarkers or as innovative therapies. In this regard, the present review aims to investigate the main functions of EVs and miRNAs derived from EVs in chronic respiratory diseases, highlighting their clinical usefulness in patients with chronic respiratory diseases.

## 2. Extracellular Vesicles

Extracellular vesicles are vesicles released into the extracellular space by most cell types, under physiological and pathological conditions and, consequently, are present in various biological fluids such as saliva, urine, bronchoalveolar lavage (BAL), plasma, sputum serum, amniotic fluid and cerebrospinal fluid (CSF) [8,9,10,11].

Until a few years ago it was thought that EVs were just a mechanism of cell rejection in order to eliminate all non-functioning cellular components. Only in recent years have they been recognized as potential means for cell-to-cell communication [12,13]. Cell-to-cell communication is an essential process for the coordination and correct organization of multicellular systems. In fact, cells communicate through various mechanisms including the release of growth factors, chemokines or small bio-active molecules and, only recently, the role of extracellular vesicles in the cellular communication mechanism has emerged. Between the cells of living beings there is a continuous exchange of information in the form of lipids, proteins and nucleic acids and, to date, we know that these molecules move, up to their destination, traveling inside extracellular vesicles, universal agents of inter-cellular communication in both normal and pathological cellular processes. Based on their origin and their size, EVs can be classified into three main classes: apoptotic bodies, microvesicles (MVs) and exosomes [4,14].

Apoptotic bodies are extracellular vesicles produced as a result of programmed cell death. They are released by the outward, blebbing or fragmentation of the plasma membrane during cell apoptosis.

Microvesicles (MVs) are smaller than apoptotic bodies, they have dimensions that can vary from 100 to 1000 nm and are produced by external budding of the plasma membrane.

Finally, exosomes are the smallest vesicles (30–100 nm), and they are formed intracellularly through three steps: (i) invagination of the plasma membrane and formation of the endosome; (ii) invagination of the endosomal membrane and formation of multivesicular bodies (MVB) which contain the intraluminal vesicles, the actual exosomes; (iii) fusion of the MVBs with the cell membrane and consequent release of the exosomes into the extracellular space [11,15,16]. Table 1 shows the three main types of vesicles and their characteristics.

After their release into the extracellular space, EVs can act both in an autocrine and paracrine way, controlling physiological processes such as development, proliferation and migration, but also pathogenetic mechanisms. In fact, they can induce a response in target cells either by releasing their cargo into the cytoplasm, which can be made up of proteins, lipids and nucleic acids (mRNA and miRNA), or by activating membrane receptors. Extracellular vesicles of various origins can be found circulating and can be isolated from different biological fluids including blood, serum, plasma, bronchoalveolar lavage fluid, urine, sputum but also supernatant of cell and tissue cultures [17]. Their composition and their cargo reflect those of the progenitor cells from which they originate; for this reason, the EVs isolated from biological fluids can represent potential biomarkers for the diagnosis and monitoring of various diseases including chronic respiratory diseases [4].

These small-enclosed vesicles can be isolated via several methods having all with advantages and limitations, which are important to consider in experimental planning. The chosen method for EV extraction should be determined based on various factors, including the type of bodily fluid, objectives of the analysis, amount of available sample and viscosity and possibility for contamination of a sample [18]. The actual gold standard is the ultracentrifugation technique that separates particles based on their size and density through sequential centrifugation steps, with increasing centrifugation forces and durations [19,20]. Density gradient centrifugation represent a density-based isolation method to isolate different EV types based on physical properties such as density and sedimentation rate. Alternative purification methods include size exclusion chromatography and ultrafiltration, which allow exosome purification based on their specific size by using nano-membrane filters [21]. New and recent methods of EVs isolation are based on the use of magnetic beads coupled with antibodies that bind specifically to a known EV-specific surface marker for separation. [21,22]. EVs characterization can be performed using several methods such as transmission electron microscopy (TEM) to visualize EVs morphology and size or nanoparticle tracking analysis (NTA) to measure concentrations and size distributions of exosomes through a laser beam that illuminates them in the sample [23,24]. To assess the protein composition of EVs, western blot (WB), antibody coated beads for flow cytometry (flow cytometry in which the vesicles have to be bound to beads due to their small size) as well as mass spectrometry-based proteomics are commonly used [25,26].

As already stated, the content of EVs includes cytokines, growth factors, proteins, lipids, DNA, mRNA and microRNA (miRNA) that can modulate the expression of target cell genes. Among these molecules, miRNAs, due to their role in regulating gene expression, are the ones that attract the attention of researchers the most [27,28].

## 3. Micro-RNA

MicroRNAs (miRNAs) are small single-stranded RNA molecules, with a length between 20 and 24 nucleotides, which play a role in regulating gene expression at the post-transcriptional level [29].

They are involved in numerous cellular processes such as cell proliferation, apoptosis and cell differentiation and can play a key role in many human diseases. 

They work by binding to complementary target sequences of messenger RNA (mRNA) in order to determine their degradation or repression of translation.

In addition, miRNAs, by binding to their target mRNA, are able to activate the recruitment and association of factors that cause the destabilization and subsequent degradation of the mRNA, resulting in a decrease in its levels of expression [30]. Currently, more than 2000 miRNAs have been identified in the human genome, which can regulate up to 30% of all protein-coding genes, despite making up only 1–3% of the human genome [31]. Therefore, these molecules represent the largest class of regulatory genes; however, the biological significance of many of them remains unknown and requires further research [31,32]. The process by which mature miRNAs are formed is quite complex and consists of numerous stages: it originates in the nucleus and is completed at the level of the cytoplasm.

Transcripts encoding miRNAs are initially transcribed by RNA-polymerase II as primary miRNAs (pri-miRNAs) with a stem-loop structure several hundred nucleotides long, with a guanosine cap in position 5’ and a polyadenylated tail in position 3’. The first miRNAs are then cut by the microprocessor, a large complex including Drosha and DGCR8, into pre-miRNAs of 70–120 nucleotides in length. The pre-miRNAs are then exported to the cytoplasm by exportin-5, where they are further cut by Dicer into mature miRNAs of 18–23 nucleotides. A cellular protein called TRBP facilitates the entry of the Dicer-miRNA complex into the complex (RISC) which contains Argonaute-2 (Ago-2), TNRC6A and other RNA-binding proteins. After incorporation of the miRNA into RISC, the two strands of the miRNA are then separated, due to the thermodynamic asymmetry of the duplex and the base pairing stability at the 5’ end and the lead strand together with the mentioned RNA binding proteins previously, the TNRC6A protein, and the Argonaute catalytic proteins, form a micro-ribonuclear protein complex (miRNP) called RISC, which binds to the target mRNA to degrade it or to inhibit its translation [33]. Several mechanisms have been proposed on the modus operandi of miRNAs, which result in the post-transcriptional repression of target mRNAs. This repression may be the result of reduced translational efficiency or an actual decrease in mRNA levels. The degree of complementarity between the miRNA and the target regions of the mRNA is essential in this choice, as it is sufficient that there is complementarity between the two transcripts to have degradation of the mRNA, while less complementarity leads to the inhibition of translation. A dysregulation of intracellular miRNA expression has been associated with numerous pathological conditions. Recent studies suggest that an altered post-transcriptional regulation is at the basis of chronic respiratory diseases. Misregulation of miRNA expression has been observed in several lung tissues and inflammatory cell types and these changes appear to be related to disease severity/risk [34]. Therefore, the alteration of miRNA expression can cause a deregulation of key genes and pathways that contribute to the development of these chronic diseases [34,35].

Many miRNAs appear to have impaired expression in chronic respiratory diseases: in particular, miR-21, miR-155 and members of the let-7 family appear to be involved in multiple distinct respiratory diseases, suggesting the importance of these miRNAs and the pathways that these miRNAs regulate lung homeostasis in global maintenance [34].

## 4. EVs-miRNA

While the majority of miRNAs are found intracellularly, significant numbers of miRNAs have been observed outside the cells, in various body fluids [36]. In this case we are talking about extracellular miRNAs which represent a form of intercellular communication through the transfer of genetic information from a donor cell to a target cell.

Thus, in addition to playing a role in the regulation of gene expression within the cells that produce them, some microRNAs can be secreted and can be found in most biological fluids (plasma, serum, saliva, urine, semen, maternal milk, amniotic fluid, cerebrospinal fluid, pleural and peritinal fluid, etc.) and their total concentration and composition varies in the different types of bio-fluids. Therefore, in clinical practice, microRNAs could be used as biomarkers for diagnosis, prognosis and response to treatment in many diseases, including chronic respiratory diseases.

The presence of microRNAs in biological fluids was first described in 2008 by Chim et al., who determined, in maternal plasma, specific placental microRNAs [37], and by Latrie et al., who detected the presence of tumor-associated microRNAs in the serum of patients with diffuse B-cell lymphoma [38]. The identification of extracellular miRNAs has generated amazement in the scientific community since it is known that biological fluids contain high amounts of RNase, enzymes responsible for the removal of exogenous nucleic acids [39]. However, to date, we know that miRNAs can circulate in body fluids complexed with Argonaute proteins, HDL (High Density Lipoprotein) particles or inside vesicles [40] and thanks to these “transport” systems they are protected from degradation by the work of ribonuclease enzymes and are very stable [40,41].

To summarize, we can therefore state that miRNAs are able to evade RNase activity in two ways:

- packed inside extracellular vesicles (EVs-miRNA);
- associated with transport proteins (EVs-free).

As described above, there are three different types of microRNA-containing vesicles: apoptotic bodies, fusion vesicles and exosomes.

However, since apoptotic bodies are the least studied vesicles in the application field, they will not be dealt with in this review, which will instead investigate the great potential of exosomes, microvesicles and EVs-miRNA as emerging research tools in the field of chronic respiratory diseases. In fact, exosomes and microvesicles, despite being very different vesicles, have common characteristics as regards their content: both types of vesicle carry RNA, microRNAs, proteins and lipids, underlining their involvement in the regulation of various biological functions. Exosomes derive from cytoplasmic multivesicular bodies whose formation and consequent release can be induced by extracellular stimuli in both physiological and pathological conditions [40].

They seem to perform various biological functions such as antigen presentation, RNA and protein transport, and cell–cell communication [42]. It is therefore possible to hypothesize that the microRNAs can be specially secreted, transported to the target cell/tissue and released through the fusion of the exosomes with the plastic membrane of the receiving cell (or through the binding to specific receptors), in order to perform their regulation function of gene expression at the level of target messenger RNAs even at “distance” from the producer cell [42,43]. After transcription in the nucleus, in fact, the miRNAs can be processed within the multivesicular bodies, containing, in addition to the miRNAs, also RNA and proteins, in the form of exosomes [44]. Once the multivesicular bodies bind to the plasma membrane, the miRNAs are released within the exosomes into the bloodstream. It is thanks to the presence of exosomes that circulating miRNAs are stable in biological fluids [44]. The second way in which circulating miRNAs can evade the activity of RNase is through association with proteins. Arroyo and colleagues were the first to hypothesize that miRNAs were not only released in biological fluids enclosed within EVs, but that they could be free from this envelope [45]. In fact, they showed that, after the ultracentrifugation of serum and plasma, a substantial amount of miRNA was present in the supernatant.

This result could be explained by hypothesizing that these miRNAs could derive from the rupture of the EVs during the ultracentrifugation steps, or from an incomplete recovery of all the vesicles present in the biological fluid analyzed. However, Arroyo and colleagues also hypothesized that these miRNAs in the supernatant could be associated with elements capable of making them stable and proggling them from the action of RNase. Therefore, with subsequent experiments they have shown that miRNAs can be associated with ribonucleoprotein complexes that guarantee their stability and protection. Further studies have shown that miRNAs in biological fluids can also be associated with AGO2 proteins, high and low density lipoproteins (HDL and LDL), and nucleophosmin protein [40,46].

However, it is still unclear whether they are packaged inside the lipoproteins together with the AGO2 proteins, or whether the mature miRNAs are associated with Apolipoprotein-I, thus forming a protein-nucleic acid complex [40,47,48,49]. Furthermore, the way in which lipoproteins are able to distribute miRNAs to target cells is not yet fully understood [47].

To date, what we know is that, not only EVs-miRNAs, but also EVs-free ones can be introduced into target cells and perform their biological function in them. 

The expression of circulating miRNAs can be easily evaluated by various methods (quantitative q-PCR, microarray and sequencing) [50,51,52]. One of the most sensitive methods for the analysis of circulating miRNAs is certainly quantitative PCR (qPCR), which involves the use of a stem-loop primer [53]. However, also the profiling of circulating miRNAs through microarrays is a technique widely used in all the studies that allow to have a quantity of starting material higher than the conventional quantity used for qRT-PCR. Furthermore, in recent years, deep sequencing has proved to be a very promising technique for the identification of novel miRNAs as biomarkers [54]. This technology has been used to compare the miRNA profile in different diseases [55,56,57]. 

There are some differences between the techniques mentioned, summarized in Table 2.

Several studies have proposed miRNAs, and in particular EVs-miRNAs, as indicators with translation to clinical practice. It is known that their transport in combination with EVs offers EVs-miRNAs an exceptional stability, sustaining their evaluation in clinical laboratories. Furthermore, as mentioned above, it is possible to analyze miRNA’s expression starting from samples obtained with minimally invasive methods and routinely used in clinical practice and through standard techniques commonly used in clinical laboratories. In addition, it is possible to explore miRNA’s profile using easily accessible methodologies such as quantitative real-time PCR, sequencing and microarrays. All these approaches allow for the detection of differences in gene expression and the association of certain molecular markers with a patient’s phenotype and disease progression. The non-invasive quantification of miRNAs has demonstrated high sensitivity and robustness as well as being economical for the clinical management of chronic respiratory diseases. 

Although the search for miRNAs as biomarkers is still in its infancy, research is investing forces and resources in the development of these molecules to guide clinical decision. Some tests are already available for clinical practice. For example, several miRNA signature-based tests are available for diagnosing different types of cancer (miRview mets and ThyraMIR). MiRNA panels are also available for risk stratification in osteoporosis (OsteomiR) and monitoring of drug effects on platelet function, reactivity and haemostasis (ThrombomiR). 

However, no tests are currently available for chronic respiratory diseases.

## 5. EVs-miRNA as Therapeutic Tools in Chronic Respiratory Diseases 

In recent decades, much research has focused on the identification of biological markers useful in clinical practice.

In 1998 the working group of the National Institute of Health defined a biomarker as a characteristic that is objectively measured and evaluated as an indicator of normal biological process, pathogenic process or pharmacologic responses to a therapeutic intervention. An optimal biomarker should be easily obtained with minimum discomfort or risk to the patient, specific, sensitive, reproducible, objective, quantifiable and economical. Even though, historically, the term biomarker includes physical traits or physiological metrics, to date, the term has expanded to molecular biomarkers. The term molecular biomarkers is a broad concept that encompasses a variety of components such as specific cells, proteins, hormones, enzymes, molecules, genes and specific mutation, RNA and microRNAs. Thus, the discovery that microRNAs are also present in biological fluids (EVs-miRNAs) has sparked great interest for their potential use as biomarkers.

Circulating biomarkers, such as EVs-miRNAs, play a significant role in clinical applications especially for the diagnosis of diseases, to monitor the therapeutic effect of a drug or to predict the progression of a disease [58].

Circulating miRNAs have many of the essential characteristics of a good biomarker: they are stable in circulation, resistant to digestion by RNase, resist extreme pH, high temperatures, prolonged storage and multiple freeze-thaw cycles [37,59]. Furthermore, to date, it is known that in many cases, changes in the expression levels of circulating miRNAs have been associated with different diseases or some biological/pathological phases.

The advantage of using miRNAs as biomarkers lies in the ease and accuracy with which they can be measured and in their extreme specificity; in fact, the miRNA profiles in the body fluids of sick people are significantly different from the profiles of healthy subjects, and some specific miRNAs are found to be enormously increased or decreased in particular diseases [60]. Furthermore, miRNAs in body fluids remain stable under various extreme conditions, such as boiling, very low or high pH, repeated freeze-thaw and storage at room temperature for a long time.

In addition, EVs are considered as valuable source for biomarkers of the pathophysiology of several diseases mainly because they are released by most cell types, representing the cell of origin, and they are present and stable in most body fluids. In consequence, microvesicles and exosomes are qualified as minimally invasive source of biomarkers for early detection, diagnosis and prognosis of many diseases.

As previously mentioned, EVs carry several types of cargo molecules and are considered to be crucial for the discovery of biomarkers for clinical diagnostics; specifically, exosomes and microvesicles loaded with specific miRNAs that could be considered as potential biomarkers for a variety of diseases, including chronic respiratory diseases, such as IPS, asthma, COPD or sleep disorders. From these considerations it follows that miRNAs in body fluids could be used as diagnostic markers for specific diseases, also because it is very easy to study the profile of the more global level of miRNA, rather than that of proteins or metabolites, thanks to the high sensitivity and speed. of profiling techniques. Blood, serum or plasma, is the most used clinical fluid sample for the identification of biomarkers since it is routinely collected in the laboratory in a rapid and minimally invasive way. However, many other fluids could be used for this purpose. Saliva, urine, spontaneous and/or induced sputum and broncho-alveolar lavage appear to be promising substrates as regards chronic respiratory diseases for the study of circulating miRNAs. In fact, the sampling methods developed in recent decades offer an innovative basis for the identification of many lung biomarkers. These techniques can be totally non-invasive, semi-invasive or invasive [61].

In this regard, we speak of non-invasive or semi-invasive sampling when we refer to:

(1) Exhaled breath condensate (EBC): Exhaled breath is saturated with water vapor which can be condensed by cooling and used to sample a wide range of mediators. EBC samples the entire respiratory tract, but newer techniques allow for fractional sampling and provide the ability to collect condensate from different parts of the respiratory tract. Collecting EBCs is a promising sampling method, but several methodological problems hinder its clinical use [61,62,63].
(2) Spontaneous or induced sputum: it is used in clinical practice for microbiological and cell counting studies, while the measurement of inflammatory biomarkers is increasingly implemented in research.

Several biomarkers have been measured in IS supernatants, notably IL-6, IL-8 and TNF-α, but methodological problems influenced the measurement making these studies inconclusive [61]. 

On the other hand, bronchial alveolar lavage (BAL) is an invasive sampling method: bronchoscopic BAL allows the study of the cellular and biochemical components present in the epithelial lining fluid [61,62,63]. One breakthrough regarding chronic respiratory diseases diagnosis using miRNA was the discovery of miRNAs in EVs [64,65].

Recently, it has been shown that many cell types of the inflammatory system, including endothelial cells, bronchial epithelial cells, dendridic cells, mesenchymal stem cells and many other cell types, are able to communicate with each other by transferring miRNA mediated by EVs [66,67,68,69,70]. Since EVs contain almost the same cell surface proteins as their cells of origin, they fuse with target cells and, once absorbed, transfer a variety of biological molecules including miRNAs [11]. This alters the biological activities of the recipient cells and influences the lung micro-environment [71,72].

In the pneumology field, the study of microRNAs and microRNAs derived from EVs has progressively grown in recent years, especially in order to search for new biomarkers with diagnostic and prognostic potential of chronic respiratory diseases and to study their possible application as a research tool in the clinical-diagnostic field.

Currently, although miRNAs and miRNAs derived from EVs have the potential to be sensitive biomarkers of various respiratory diseases, further studies are needed to empower the associations identified between biomarkers and these diseases. 

As previously discussed, miRNA and EVs-miRNA currently seem to have a role as potential indicators of the clinical management of the chronic respiratory pathologies. Much progress has been made, but, to date, we are still far from their use in clinical routine.

At this regard, further efforts are needed to address the current gaps which should be overcome for the clinical application of miRNA and EVs-miRNA in patients with chronic respiratory diseases. 

In particular, it is known that the expression of miRNA or EVs-miRNA is a dynamic parameter that depends on the stage of the disease, on age, sex and other clinical characteristics of patients. In addition, the presence of specific SNPs (single-nucleotide polymorphism) can influence the expression of the miRNA’s profile. Therefore, the interval of its concentration can be ethnic-specific and significantly vary among patients.

Based on the information that we actually have, the use of molecular information obtained by miRNA and EVs-miRNA analysis looks promising. However, further studies are needed that take into account the current limitations of EVs-miRNAs to clarify their clinical application. 

The role of EVs-miRNAs in the prognosis of chronic respiratory diseases is still unknown and this research field should be explored in the near future.

This review is therefore focused on miRNAs contained within extracellular vesicles (EVs) as biomarkers in early diagnosis and as therapeutic targets for chronic respiratory diseases. 

**Figure 1:**
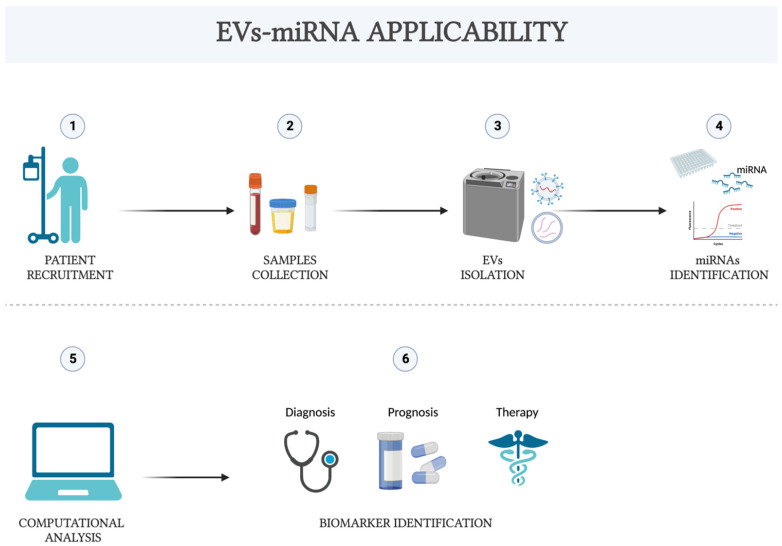
Flowchart of the potential applications of EVs-miRNA from patient selection to biomarker identification.

Figure 1 represent the potential applicability of EVs-miRNA as diagnostic, prognostic and therapeutic biomarker.

### 5.1. EVs-miRNAs in Airflow Obstruction

Obstructive diseases are lung diseases characterized by chronic inflammation with different etiopathogenetic, clinical and functional characteristics. The inflammation due an obstruction of the airways and consequent difficulty in the exhalation phase [73].

The main types of obstructive pulmonary diseases are:

- Chronic obstructive pulmonary disease (COPD), a disease of the respiratory system characterized by irreversible obstruction of the airways [74].
- Asthma, a condition characterized by the restriction of the airways, usually reversible, in response to stimuli such as allergy or infection [75].

#### 5.1.1. COPD and EVs-miRNAs

EVs-derived miRNAs in COPD have great potential for understanding disease pathogenesis and identifying important new biomarkers. In this section, we briefly present the available research on EVs-miRNAs in the pathogenesis and diagnosis of COPD to identify existing knowledge and support further research in this field.

Chronic obstructive pulmonary disease (COPD) is defined by the World Health Organization as not a single disease, but as a general term used to describe chronic lung diseases that cause restrictions in the flow of air to the lungs [74]. COPD is in fact a set of diseases of the respiratory system characterized by an irreversible obstruction of the airways, of variable extent depending on the severity, usually progressive, associated with a state of chronic inflammation of the lung tissue. The long-term consequence is a real remodeling of the bronchi, which causes a significant reduction in respiratory capacity.

This clinical picture is aggravated by the increased predisposition to respiratory infections of viral, bacterial or fungal origin. There is currently no effective cure, but several treatments are available to control symptoms and to avoid dangerous complications. On the other hand, prevention is fundamental to minimize risk factors (cigarette smoke, atmospheric pollution, etc.) [3,76].

Indeed, smoke and other inhaled irritants cause an inflammatory response in the peripheral airways and lung parenchyma [67]. The origin of COPD could therefore be due to an abnormal inflammatory response combined with oxidative stress, both caused by inhaled harmful agents.

Inflammatory and structural changes in the airways increase with disease severity and persist with smoking cessation. Inflammation of the respiratory tract of COPD patients appears to be an amplification of the normal inflammatory response of the respiratory tract to chronic irritants such as cigarette smoke. Lung inflammation is further amplified by oxidative stress and an excess of proteinase in the lung. Together, these mechanisms lead to the characteristic pathological changes in COPD [77,78].

COPD is a disease that is often diagnosed in an advanced stage of the disease, when patients are experiencing a substantial deterioration in their quality of life. Currently, spirometry is the most widely used diagnostic approach to assess the state of the disease and the only lung function test capable of detecting the severity of the disease. However, as mentioned, the functional damages unfortunately only show when the pathology is in an advanced form and not in the early stages of the disease.

In this regard, however, some inflammation markers seem to play a key role in early diagnosis, in assessing prognosis and in assessing the ability to respond to therapy or the effectiveness of the therapy itself.

To date, the only widely used biomarkers for COPD are lung function tests, FEV1 and FEV1/FVC combined with systemic inflammation biomarkers: fibrinogen, interleukin 6 (IL-6), interleukin 8 (IL-8) and protein C reactive (CRP).

Although FEV1 and FEV1/FVC and markers of inflammation are easy to obtain and reproducible, they do not inform about underlying disease activity, do not separate COPD phenotypes, are not COPD specific, and do not respond to some therapies that clearly improve survival [62].

Therefore, new biological markers, which reflect the presence, severity or state of a disease, easily measurable with standard techniques, easily reproducible, highly sensitive and specific and above all representative of the various stages of the disease, are needed to give consistent help to the medical practice in achieving a complete and timely evaluation of patients, for a better management of diagnosis, response to therapy and follow-up.

Recently an interesting approach with promising results seems to be provided by the study of extracellular vesicles and miRNAs derived from extracellular vesicles in order to discern patients with and without COPD which, integrated with the analysis of other biomarkers and clinical parameters, is believed to be able to improve, in the future, the ability of doctors to monitor this pathology.

Numerous studies have reported various circulating miRNAs involved in the development and progression of COPD [9,79,80,81].

Firstly, Fujita et al. reported that intercellular communication and pathogenesis of COPD can be modulated by miRNAs through EV-mediated miRNA transfer [68]. In particular, they observed that miR-210 expression was significantly higher in extracellular vesicles released from primary human bronchial epithelial cells (HBEC) after exposure to cigarette smoke and was able to promote fibrosis of the airways in the pathogenesis of COPD [68]. They therefore showed that exposure to stressors such as cigarette smoking changes the composition of EVs, potentially counteracting pathological disorders such as airway remodeling in COPD.

Secondly, Serban et al. investigated the characteristics of extracellular vesicles (microvesicles and exosomes) released by epithelial cells following exposure to cigarette smoke. They reported that the released vesicles were mostly exosomes to be transferred to macrophages, secreted thanks to the action of the ceramide sphingomyelinase synthesis enzyme which, following exposure to smoke, showed a reduced expression within them of specific miRNAs including let-7d, miR-191, miR-126 and miR125a, promoting the clearance of apoptotic cells [67].

Only recently has it been discovered that exosomal miR-21, derived from bronchial cells, is responsible for the differentiation of myofibroblasts in response to exposure to cigarette smoke. Elevated levels of exosomal miR-21 are in fact elevated in the serum of smokers and COPD patients [82].

A further study made it possible to identify EVs-miRNAs as diagnostic biomarkers to distinguish patients with lung cancer and patients with COPD, as well as to determine the differences between smokers suffering from these pathologies [83]. In this study, O’Farrell et al. have demonstrated the applicability of EVs-miRNAs as a screening tool for patients with COPD or lung cancer. For the study they isolated extracellular vesicles from the plasma of: (i) 20 healthy subjects who had never smoked, (ii) 20 healthy smokers, (iii) 20 participants with a history of smoking and diagnosed with lung cancer, and (iv) 20 participants with clinically stable COPD, after which they extracted the RNA and analyzed a series of miRNAs derived from the EVs. A total of 15 miRNAs were found to be differentially expressed between lung cancer and healthy non-smoking participants, 26 miRNAs were significantly downregulated between lung cancer and healthy smokers and, finally, 14 miRNAs appeared significantly dysregulated between lung cancer and stable participants. with COPD. Thanks to this study, O’Farrell et al. have succeeded in demonstrating that the miRNAs contained in plasma EVs contain key biological information capable of allowing the discrimination of patients with COPD and patients with lung cancer, highlighting the potential of EVs-miRNAs as specific signatures for pathological states and underlining their potential for biomarkers, thanks to their involvement in specific stages of the disease [83].

Finally, we know that skeletal muscle weakness is an important systemic complication of COPD capable of influencing the motility and mortality of people affected by this disease. Burke et al., in this regard, evaluated the role of exosomal miRNAs in patients with COPD and skeletal muscle weakness [84]. They isolated exosomal miRNAs from the serum and bronchoalveolar lavage (BAL) of four COPD patients, showing that one exosomal miRNA was upregulated in the serum of COPD patients and four exosomal miRNAs were downregulated in the BAL of COPD patients. Furthermore, the in silico analysis they conducted indicated numerous genes as potential targets of these miRNAs, including S6K, involved in the mTORC1 signaling pathway which acts as a key regulator of skeletal muscle wasting. Thanks to these promising results, Burke et al. thus demonstrated that exosomal miRNAs play a critical role in skeletal muscle wasting in COPD patients and, thanks to this role, could be used as diagnostic and prognostic biomarkers for the detection, treatment and monitoring of patients with this disease [84].

In conclusion, COPD is known to be a multifactorial disease, in which many factors, such as cigarette smoking, play a key role. All these factors are in fact able to exert their effect by acting on different pathways involved in the pathogenesis of COPD. Among the molecules involved in the development of the disease, miRNAs and exosomes have emerged as important actors. A modulation of miRNAs by targeting various cellular and molecular pathways involved in COPD could contribute to the development and progression of the disease. Novel biomarkers related to prognosis, diagnosis, therapy and therapeutic response are needed to monitor disease progression in COPD patients and help physicians in treatment decisions. Among these biological markers, miRNAs derived from extracellular vesicles appear to be promising new tools to be used as diagnostic and therapeutic biomarkers in the treatment of COPD. Many of the studies mentioned are listed in Table 3.

#### 5.1.2. Asthma and EVs-miRNAs

Asthma is a chronic disease characterized by recurrent attacks of dyspnea and wheezing, which vary in severity and frequency from person to person [75]. It is a complex disease that manifests itself through chronic inflammation of the airways; the inflammation generates an increase in bronchial responsiveness which, in turn, causes recurrent episodes (so-called ‘asthma attacks’) of respiratory crises, chest tightness and cough. During attacks, which can be sudden or gradual, symptoms and respiratory function worsen. If not treated properly, the attacks can be very serious and even fatal. Identifying chronic inflammation as the key point in defining the disease, as has happened in recent years, has had important repercussions at both a diagnostic and therapeutic level [75]. To date, doctors have a limited choice of anti-inflammatory and bronchodilator treatments for asthma and there is no cure for this condition. Further studies are therefore needed to better understand the mechanisms underlying the disease and to identify new possible molecular targets. Several studies have demonstrated the important role played by microRNAs in asthma, in particular it has been observed that over 100 miRNAs seem to have a differential expression in asthmatic patients compared to healthy controls [75,85,86,87,88,89]. It is also known that some miRNAs are secreted in the exosomes that are released by the cells associated with asthma following various non-specific stimuli and, to date, these exosomal miRNAs are among the main players in the pathogenesis of the disease. In fact, emerging studies have shown that exosomes and exosomal miRNAs released by cells associated with asthma pathology such as mast cells, eosinophils, neutrophils and T lymphocytes, can participate in intercellular crosstalk, act as intercellular mediators and allow the exchange of information, contributing to airway hyperactivity (AHR), inflammation and remodeling of the airways [28,90,91,92,93,94]. In order to identify the exosomal microRNAs associated with asthma, several studies grouped in Table 4 were conducted. Sinha et al. were among the first to demonstrate that exosomes are present in the exhaled breath condensate (EBC) of asthma patients and that these vesicles contain most of the miRNAs detectable in the exhaled breath condensate [95]. Levanen et al. then demonstrated that eight exosomal miRNAs, respectively, let-7a, miRNA-21, miRNA-658, miRNA-24, miRNA-26a, miRNA-99a, miRNA-200c and miRNA-1268, show substantial differences in expression in the BAL of patients. with mild intermittent asthma compared to a group of healthy control patients [96]. They analyzed exosomal miRNAs first by microarrays and the selected results were validated by qRT-PCR. Most of the exosomal miRNAs found to be altered showed a lower expression in asthmatic subjects than in healthy subjects, suggesting their involvement in the regulation of IL-13.

Similarly, Gon et al. conducted experiments on animal models and showed that, also in this case, the exosomes secreted by the cells of the airways in mice exposed to house dust mites compared to control mice had an upregulated expression of some miRNAs contained within them [97]. In particular, through microarrays and quantitative analysis of the polymerase chain reaction (qRT-PCR), they observed a constantly upregulated expression of three exosomal miRNAs, respectively, miR-1827, miR-346 and miR-574-5p. The authors of this study also observed that, in mice exposed to house dust mites, the amount of exosomes released from the airways seemed to decrease following treatment with the sphingomyelinase inhibitor GW4869. According to this study, treatment with this inhibitor could reduce the amount of Th2 cytokines and eosinophils in the BAL and reduce the accumulation of eosinophils in the walls of the airways and mucosa, relieving inflammation of the allergic airways. However, the authors did not further investigate whether these altered exosomal miRNAs played a crucial role in allergic airway inflammation [97].

Subsequently, Qiao Y. et al conducted a study in rats and identified 23 exosomal serum miRNAs with impaired expression among rats with airway inflammation caused by zinc oxide nanoparticles and control rats. The authors have shown that such exosomal miRNAs could be involved in lung inflammation; however further studies are needed to support this hypothesis and, currently, it is not entirely clear how these particles contribute to the pathology of asthma [98].

Gutierrez et al. characterized the profiles of miRNAs secreted in extracellular airway vesicles by measuring the expression of nasal exosomal miRNAs in children with or without rhinovirus (RV) infection, the most common cause of asthma exacerbations and one of the major risk factors for the development of pathology. They identified four exosomal miRNAs (hsa-miR-630, hsa-miR-302d-3p, hsa-miR-320e and hsa-miR-612) that were found to be constitutively expressed in nasal airway secretions in infants with RV. It was also found that exosomal miR-155 was only detectable in RV-infected children [99]. 

All the studies mentioned above demonstrate the role of miRNAs and in particular of the miRNAs contained within exosomes in the development of asthma; however, the functioning of exosomal miRNAs in the disease is not yet fully understood. Therefore, further studies are needed to clarify the potential mechanisms involved and the targets and signaling pathways related to asthma that these exosomal miRNAs control, in order to develop effective therapeutic strategies and new diagnostic methods. Many of the studies mentioned are listed in Table 4.

### 5.2. Sleep Disorders and EVs-miRNAs

The term respiratory disorders in sleep (SDB) is a general term used to indicate a range of conditions that cause abnormal breathing during sleep, in many cases associated with narrowing or complete and/or partial obstruction of the upper airways. The most common of these is obstructive sleep apnea (OSA) [100]. OSA is characterized by episodes of obstruction of the upper airways: these are defined as apneas, if the airways are completely occluded, and hypopneas, if the occlusion is only partial. It is caused by increased collapsibility of the upper airways, along with insufficiency or loss of muscle capacity to dilate the upper airways that promotes narrowing or closing of the pharynx (apneas or hypopneas), thus leading to a decrease in oxyhemoglobin saturation and an increase in the partial pressure of carbon dioxide in arterial blood [101]. Obstructive sleep apnea, the most severe form of sleep breathing disorder, is characterized by intermittent sleep hypoxia (IH), sleep fragmentation and episodic hypercapnia. Symptoms include excessive daytime sleepiness, restlessness, snoring, recurrent awakening and morning headaches. Currently, the diagnosis is based on sleep history and polysomnography. Treatment consists of the application of continuous positive pressure ventilation, oral appliances and, in refractory cases, surgery [102]. Generally, with treatment, the prognosis is good. However, most cases are not diagnosed and treated in time and are often associated with hypertension, atrial fibrillation and other arrhythmias, heart failure, and injury or death from motor vehicle and other accidents resulting from hypersomnolence.

OSA is in fact associated with an increased risk of morbidity and mortality affecting the cardiovascular, metabolic and neurocognitive systems and, more recently, with non-alcoholic fatty liver disease and cancer-related deaths [103,104,105].

Substantial variability in OSA outcomes suggests that genetically determined and environmental factors and lifestyle influence phenotypic susceptibility to OSA. Furthermore, OSA and obesity often coexist and manifest the activation of shared molecular mechanisms of end organ damage which, if properly identified, can represent potential therapeutic targets.

A challenge in the diagnosis and prognosis of this pathology is the development of new clinically relevant biomarkers in biological fluids using non-invasive diagnostic tests. The use of new biomarkers, related to the severity of the disease, is therefore necessary in order to characterize the pathology allowing a simplified and reliable diagnostic screening and helping the doctor in the selection of patients for diagnosis and treatment.

In this regard, circulating miRNAs have been proposed as interesting candidates as both diagnostic and prognostic biomarkers in OSA, whereby the dysregulation of specific miRNAs in response to genetic or environmental factors could contribute to prognosis, diagnosis, therapy and response, and assisting physicians in monitoring disease progression and making therapeutic decisions [106].

The ability of exosomes to transport a cargo to distant recipient cells has, in fact, led in recent years to consider exosomes, or extracellular vesicles in general, as important mechanistic determinants in various pathologies.

Since intermittent hypoxia (IH), characteristic of obstructive sleep apnea, is an important risk factor for cardiovascular complications and since more and more studies have revealed alterations in the contents of extracellular vesicles, Li et al. have recently identified a unique cluster of exosomal miRNAs, which play a pathological role in cardiovascular complications, in humans and rodents exposed to intermittent hypoxia and in patients with OSA and various pathological phenotypes [107].

For this purpose, they identified 63 miRNAs differentially expressed in the EVs derived from cardiomyocytes exposed to IH compared to the control group and demonstrated that the EVs derived from cardiomyocytes treated with IH are able to inhibit the Akt/eNOS signaling pathway causing endothelial relaxation. From these observations, we can therefore deduce that the IH is able to alter the composition of the miRNAs carried in the EVs released by the myocardial cells and that the EVs released by the cardiomyocytes play a fundamental role in the endothelial function in conditions of IH, thus explaining the endothelial dysfunction characteristic of patients with OSA [107]. In addition, numerous studies have recently been published focusing on plasma and exosomal miRNAs in OSA.

Khalyfa et al. have conducted a series of studies to investigate whether plasma miRNAs in children with OSA can undergo variations based on endothelial functional status [108].

Subsequently, the same researchers examined a specific panel of miRNAs in children with OSA and obesity, both conditions associated with an increased risk of endothelial dysfunction, and observed a discrepancy in the expression of some miRNAs in subjects with endothelial dysfunction compared to those with normal endothelial function. They then expanded on these findings for children with OSA and obesity by also analyzing exosomal miRNA load rather than just plasma miRNAs. This study allowed for the identification of five differentially expressed exosomal miRNAs [109].

In particular, the expression of miR-630 was significantly reduced in children with endothelial dysfunction, regardless of whether they had OSA, obesity or both. They showed that miR-630 is able to regulate gene targets in endothelial cells including NRF2-mediated stress responses, AMP kinase and tight junction signaling pathways, bringing to light a new role of exosomal miRNA-630 as a presumed mediator of vascular function and cardiovascular disease risk in children with OSA and/or obesity, and identifying this miRNA as a possible oxidation therapeutic target [109].

On this subject, Khalyfa and Gozal also recently published a review in which they explain the composition and functional properties of exosomes, focusing on their potential as diagnostic biological markers of cardiovascular risk in children suffering from previous conditions associated with a higher prevalence of cardiovascular diseases, such as obesity and sleep breathing disorders [110].

In parallel, Khalyfa et al. conducted evaluations of plasma-derived exosomes in healthy adults exposed to experimental intermittent hypoxia. The exosomes, following exposure to IH, have shown a greater ability to interfere with endothelial function in naive endothelial cells and have been shown to be able to recruit the expression of adhesion molecules that promote the adhesion of monocytes. In addition, they demonstrated the presence of differentially expressed miRNAs in the exosome load and sought to provide an explanation for the widespread transcriptomic effects of these exosomes on endothelial cells. All subjects were then submitted to a normoxic recovery of a similar duration to exposure to IH and, following this recovery, the content and functional properties of the exosomes studied returned to baseline [111].

Almendros et al. observed that IH, which mimics OSA in vitro, is able to induce changes in the biological characteristics of some solid tumors in vivo [112]. Following this, they proposed an experimental model of IH in mice and performed experiments similar to those just described, conducted by Khalyfa et al. in a human. Almendros and researchers have identified a number of exosomal miRNAs capable of promoting changes in the carcinogenic properties of some cancer cells [113]. Specifically, they identified 11 differentially expressed miRNAs in mice exposed to IH and identified their gene targets in TC1 tumor cells. All of these studies of OSA exosomes in human and mouse models, using intermittent hypoxia as a tool to mimic the characteristic apneas of patients with OSA, provide compelling justification for further studies to characterize how the IH alters the generation and release of exosomes, as well as the influence of IH on the loading and function of exosomes. Based on these considerations, it is evident that exosomes, miRNAs and EVs-miRNAs are slowly developing and could provide opportunities to explore new pathogenesis mechanisms of obstructive sleep apnea that could then also be exploited as clinical tools. We currently know that biomarkers, including DNA, RNA, proteins and metabolites, and miRNAs, can reflect an individual’s health or disease status [114].

Based on their usefulness in practice, biomarkers can provide information on the diagnosis, prognosis, regression or response to treatment of a disease [115].

Currently, invasive and non-invasive procedures often used to identify biomarkers and the field of sleep in general, and that of OSA in particular, require the development and validation of new biomarkers [116]. Considering therefore that practically all body fluids contain exosomes and considering the ability of exosomes to carry a load to distant recipient cells, these vesicles and the miRNAs contained within these vesicles appear to be important new biomarkers for various diseases and in particular for sleep disorders and all associated morbidity [117,118,119]. Further studies are needed on the biology of exosomes and EVs-miRNAs in the context of IH and OSA to improve understanding of the disease and its consequences. Table 5 summarizes most studies in this regard.

### 5.3. Idiopathic Pulmonary Fibrosis and EVs-miRNAs

Idiopathic pulmonary fibrosis (IPF) is a chronic, age-related, progressive and fatal lung disease characterized by damage and apoptosis of alveolar epithelial cells, epithelial-mesenchymal transition (EMT), increased proliferation of fibroblasts, and accumulation of extracellular matrix (ECM) in the interstitial tissue [120]. Compared to other respiratory diseases, there is much less research on new pharmacological treatments and biomarkers useful for early diagnosis to improve and extend life expectancy by slowing the rapid progression of the disease (the prognosis of IPF is poor and patients with IPF show a median survival of 2–3 years) [121].

It has been seen that epithelial cell senescence could promote IPF, as these cells release EVs which reduce the reparative activity of stem cells [122,123]. Chanda et al. showed that electric vehicles of fibroblasts carry fibronectin and other ECM proteins that enhance fibroblast invasion [124]. Another report showed that fibroblasts produce extracellular vesicles containing antifibrotic prostaglandins that can inhibit myofibroblast differentiation, demonstrating the involvement of IVs in IPF and their possible use as shuttles for the delivery of targeted antifibrotic therapies [125].

Several studies have shown the possibility of isolating electric vehicles from bronchoalveolar lavage (BAL) to use them as reliable biomarkers with respect to invasive lung tissue biopsies.

Martin-Medina and coworkers demonstrated that electric vehicles isolated from IPF contain WNT5A, a protein associated with a signaling pathway that contributes to the disruption of lung epithelial cell homeostasis during IPF. They found increased levels of WNT5A in the electric vehicles of people with IPF compared to non-IPF controls [126]. Zhu et al. found a high level of miR-204-5p contained in BAL-derived exosomes of rats with pulmonary fibrosis; they also demonstrated that exosomal miR-204-5p in vitro from BAL represses autophagy to promote lung fibroblast proliferation by regulating AP1S2 expression [127]. Another study evaluated exosomal miRNA signature in BAL from patients with IPF and healthy participants, showing that miR-125b, miR-28, miR-21, miR-100, miR-140-3p and miR-374b were upregulated in patients with IPF, while let-7d, miR-103, miR-26 and miR-30a-5p were downregulated. Furthermore, Liu B. et al. demonstrated that miR-30a-5p overexpression attenuated transforming growth factor-β1 (TGF-β1)-induced upregulation of TAB3, α-SMA and fibronectin expression in 293T cells. Therefore, probable exosomal miR-30a-5p isolated from BALF could be a potential biomarker for the diagnosis of IPF and in vitro increase in miR-30a-5p levels could be a potential strategy for IPF management [128].

In our previous research we analyzed exosome-derived miRNA expression levels in the serum of patients with IPF showing a statistically significant decrease in miR-16 and let-7d compared to healthy subjects and a potential antifibrotic effect of let-7d [8]. Furthermore, in our other study, we characterized the expression of exosome surface epitopes from the serum of patients with IPF, demonstrating that CD19, CD69, CD8 and CD86, CD209, Cd133/1, MCSP and ROR1 were upregulated in patients with IPF. with respect to controls. In contrast, CD42a was lower in subjects with IPF than in controls [129].

In a recent study, increased expression of EVs-derived miR-21 was found in serum samples in both mouse models with IPF and patients with IPF, demonstrating a possible correlation with IPF progression and mortality. These results suggest that serum-EV-miR-21-5p could be a suitable diagnostic marker for IPF [130].

Further studies investigated the correlation between IV-derived miRNAs and IPF progression of IPF. Njock M-S et al. found higher expression levels of seven miRNA contents in sputum-derived IPF exosomes compared to controls and a negative correlation between miR-142-3p and lung function as assessed by DLCO/VA, a marker of function alveolo-capillary. These results suggest that those miRNAs may be promising biomarkers but may be involved in disease [131].

Guiot et al. purified exosomes from plasma and sputum of subjects with IPF and healthy controls to study the impact of exosomal IPF miRNAs on IPF progression. They found significant upregulation of exosomally derived miR-142 in both plasma and sputum of IPF compared to healthy subjects and demonstrated that exosomally derived miR-142 probably plays an antifibrotic role by reducing TGFβ-R1 in the alveolar epithelium lung cells and fibroblasts [132].

Previous studies indicated that EV-MSCs inhibited pulmonary fibrosis by transferring microRNAs.

Zhou et al. showed that miR-186 from human bone marrow mesenchymal stem cell-derived extracellular vesicles (BMSC-EVs) suppressed the expression of SOX4 and its downstream gene Dickkopf-1 (DKK1) by blocking the activation of fibroblasts highlighted its possible role as a therapeutic target for IPF [133]. Furthermore, the miR-29 provided by EV derived from human BM-MSCs decreases the expression of frizzled 6 (FZD6) by repressing the proliferation of fibroblasts [134]. Gao and coworkers found that extracellular vesicles derived from adipose mesenchymal stem cells suppressed pulmonary fibrosis by inhibiting TGF-bRI (transforming growth factor-β type I receptor) by transferring let-7d-5p into alveolar epithelial cells [135].

The discovery of biomarkers in IPF is a clinically important field and EVs and EV-miRNAs may eventually be used for the pathogenesis, diagnosis and treatment of lung diseases [136]. Table 6 showed the main studies above mentioned.

## 6. Conclusions

The extracellular vesicles, and in particular the exosomes, due to their cellular origin and their role in both physiological and pathological conditions in recent years, have significantly changed many areas of clinical science. In fact, thanks to their protein and nucleic acid content, which reflects the nature and state of the cells that release them, extracellular vesicles are considered a rich source of information. The molecules carried by the EVs are therefore useful both for diagnostic purposes and for the monitoring of therapeutic treatments; moreover, their content, which is well protected by a lipid bi-layer which confers a high degree of stability, includes some biomarkers that would otherwise be poorly detectable.

Much progress has been made in the pneumology field, where many microRNAs contained in EVs have been associated with the progression of various respiratory diseases.

For example, Rabinowits and colleagues demonstrated the significant difference in exosome and miRNA levels between lung cancer patients and healthy patients, suggesting that exosome-borne microRNAs could be useful for developing screening tests for this neoplasm [137].

It is known that EVs have a heterogeneous cargo capable of altering with the state of the disease, which makes these particles new potential diagnostic biomarkers for various diseases or to evaluate the efficacy of drugs. EVs can also deliver specific mRNAs, miRNAs and proteins directly to recipient cells and alter their function, suggesting they could be an effective tool for targeting therapy to specific cells and organs.

Based on the present evidence, the exosomes and miRNAs contained within the EVs provide new diagnostic biomarkers for a wide range of lung diseases and can also be used for therapeutic interventions.

## Figures and Tables

**Table 1:** Main types of extracellular vesicles.

Vesicle	Size	Origin	Markers	Function
Exosome	30–100 nm	Endocytic origin	Alix, Tsg101, Tetraspanins (CD81, CD63, CD9), flotillin	Cell–cell communication.
Microvesicle	100–1000 nm	Plasma membrane budding	Integrins, selectins, CD40	Cell–cell communication.
Apoptotic body	1000–4000 nm	Blebbing	Annexin V, TSP, C3P, exposed phosphatidylserine	Product of programmed cell death. Facilitate clearance of apoptotic cells.

**Table 2:** Main techniques for the quantification of miRNAs in body fluids.

Technique	Advantages	Disadvantages
RT-PCR	High sensibilitỳ and specificity; Suitable for quantitative studies; Simple to perform, it does not require equipped laboratories or qualified personnel.	Much time is needed to process a limited number of samples; It allows the identification of known/selected mi-RNAs only.
Microarray	It allows the processing of a large number of samples in a very short amount of time; Not very accurate for quantitative studies.	It allows the identification of known/selected mi-RNAs only.
Deep sequencing	Identifies unknown miRNA; Identifies splice variants (isoMir); Distinguishes miRNA con similar sequences.	High cost; Specific equipment and specialized personnel are required; High amount of biological material required.

**Table 3:** miRNA profiles in EVs from COPD patients.

References	Year of Publication	Study Design	Samples	Altered miRNA	Results
Fujita et al. [68]	2015	Study of EVs derived miRNAs in HBEC from COPD subjects after exposure to cigarette smoke.	HBE cells	miR-210	miR-210 expression was greater in HBEC cells isolated from lung samples.
Xu et al. [82]	2018	Analysis of the role of exosomal miR-21 in the dysfunctional epithelium-fibroblast cross-talk caused by cigarette smoke.	SERUM, HBE cells	miR-21	miR-21 was high in serum of smokers and COPD patients.
Serban et al. [67]	2016	Investigation of extracellular vesicles following exposure to cigarette smoke.	PLASMA, human lung endothelial cells	Let-7d, miR-191, miR-126, miR-125a	Let-7d, miR-191, miR-126 and miR-125a were upregulated after being exposed to cigarette smoke.
O’Farrell et al. [83]	2021	Study of extracellular vesicle miRNAs in healthy non-smokers, healthy smokers, lung cancer and COPD patients.	PLASMA	up-regulated: miR-485, miR-27a-3p, miR-106b-3p down-regulated: miR-205-5p, miR-199a-5p, miR-497-5p.	Identification of some dysregulated EV miRNAs which could discriminate between groups of lung cancer cases compared to healthy non-smokers, healthy smokers and stable COPD cases.

**Table 4:** miRNA profiles in EVs from asthmatic patients.

References	Year of Publication	Study Design	Samples	Altered miRNA	Results
Sinha et al. [95]	2013	Study of approximately 1000 miRNAs from 10 subjects with asthma and 10 healthy subjects.	EBC	643 miRNA among which: let7, miR181c, miR-1307, miR-574-5p, miR-516a-5p, miR-42, ecc.	A total of 643 miRNAs were found in EBC with a significant difference in expression between the two groups. Furthermore, their presence has been demonstrated in vesicles capable of guaranteeing their stability in body fluids.
Levänen et al. [96]	2013	Analysis of exosomal miRNAs in a group of asthma patients vs. a group of healthy controls.	BAL	Let-7a, miR-21, miR-658, miR-24, miR-26a, miR-99a, miR-200c, miR-1268	There are many differences in exosomal miRNA profiles between healthy subjects and patients with unprovoked, mild, stable asthma.
Gon et al. [97]	2017	Exosomal miRNA analysis in a group of asthmatic mice vs. a group of healthy mice.	BAL	miR-1827, miR-346, miR-574-5p.	The sorting of miRNA into EVs would be involved in the pathogenesis of allergic airway inflammation.
Qiao Y et al. [98]	2018	Serum exosomal miRNA analysis in rats with airway inflammation caused by zinc oxide nanoparticles and control rats.	SERUM	up-regulated: miR-134-5p, miR-207, miR-465-5p. down-regulated: miR-30b-5p, miR-19a-3p, miR-130a-3p	Serum exosomal miRNAs are involved in lung neutrophilic inflammation induced by zinc oxide.
Gutierrez et al. [99]	2016	Characterization of nasal exosomal miRNAs in children with or without rhinovirus (RV) infection.	Nasal airway secretions	miR-630, miR-302d-3p, miR-320 e miR-612	Exosomal miR-155 is overexpressed in the upper airways of RV infected individuals.

**Table 5:** miRNA profiles in EVs from OSA patients.

References	Year of Publication	Study Design	Samples	Altered miRNA	Results
Khalyfa et al. [109]	2016	Circulating exosomal miRNAs analysis in children with OSA.	PLASMA	down-regulated: miRNA-16–5, miRNA-451a, miRNA-5100, miRNA-630. up-regulated: miRNA-4665–3p	Identification of a cluster of plasma-derived exosomal microRNAs to distinguish between normal and abnormal endothelial function in children with obstructive sleep apnea or obesity.
Khalyfa et al. [111]	2016	Study of microRNA from plasma exosomes in subjects exposed to intermittent hypoxia.	PLASMA	miR-4649-3p, miR-4436b-5p, miR-483-3p, miR-1202, miR-4505	Intermittent hypoxia alters exosome cargo.
Almendros et al. [114]	2016	Analysis of exosomal miRNA under intermittent hypoxia conditions.	Human and mice PLASMA	miR-6418-5p, miR-6366, miR-5100, miR-451a, miR-5113, miR-671-5p, miR-709, miR-3082-5p, miR-882, miR-92-3p, miR-2137	Circulating exosomes released under intermittent hypoxia conditions promote oncogenesis.

**Table 6:** miRNA profiles in exosomes and EVs from IPF patients.

References	Year of Publication	Study Design	Samples	Altered miRNA	Results
Lacedonia et al. [8]	2021	Characterization of exosomal derived miRNAs in IPF patients and healthy controls.	SERUM	down-regulated: let-7d, miR-16	Let-7d and miR-16 were found decreased in IPF subjects compared to normal subjects.
Zhu et al. [127]	2021	Analysis of exosomal miRNAs in rat with pulmonary fibrosis.	BAL	miR-204-5p	miR-204-5p induce the progression of pulmonary fibrosis in rats.
Liu et al. [128]	2018	Analysis of miRNA from exosomes useful as potential biomarkers for idiopathic pulmonary fibrosis.	BAL	up-pregulated: miR-125b, miR-128, miR-21, miR-100, miR-140-3p, miR-374b. down-regulated: let-7d, miR-103, miR-26 and miR-30a-5p.	Identification of miRNA biomarkers associated with idiopathic pulmonary fibrosis.
Makiguchi Y et al. [130]	2016	Serum extracellular vesicles miRNA evaluation as putative prognostic biomarkers of idiopathic pulmonary fibrosis.	SERUM	miR-21-5p	Serum extracellular vesicles miR-21-5p seems a potential prognostic biomarker for IPF.
Guiot et al. [132]	2019	To assess whether the IPF-related exosomal miRNA play a role in the progression of pulmonary fibrosis.	SPUTUM, PLASMA	miR-142-3p	Exosomally derived miR-142 probably plays an antifibrotic role by reducing TGFβ-R1.
Zhou et al. [133]	2021	Study of exosomal miRNA to improve the therapeutic approach of idiopathic pulmonary fibrosis.	MESENCHYMAL STEM CELLS	miR-186	miR-186 in extracellular vesicles from bone marrow mesenchymal stem cells relieves idiopathic pulmonary fibrosis,
Wan et al. [134]	2020	Evaluation of cargo from mesenchymal stem cells-derived EV (extracellular vesicles) in IPF.	MESENCHYMAL STEM CELLS	miRNA-29b-3p	miRNA-29b-3p from mesenchymal stem Cell-derived extracellular vesicles decrease the FZD6 expression repressing the fibroblast proliferation.

## Data Availability

The data presented in this study are available on request from the corresponding author.
